# Electrochemically Controlled Raman Response in Electropolymerized Polymethylene Blue–Gold Nanoparticle Films on Screen-Printed Carbon Electrodes

**DOI:** 10.1021/acsomega.6c00888

**Published:** 2026-03-30

**Authors:** Azeneth Borja, Fernando A. Basso, Henry S. Kavazoi, Christopher M. A. Brett, Priscila Aléssio, Cibely S. Martin

**Affiliations:** 1 School of Engineering, São Paulo State University (UNESP), Ilha Solteira, SP 15385-007, Brazil; 2 School of Sciences and Technology, São Paulo State University (UNESP), Presidente Prudente, SP 19060-080, Brazil; 3 Department of Chemistry, CEMMPRE, ARISE, Faculty of Sciences and Technology, University of Coimbra, Coimbra 3004-535, Portugal

## Abstract

Hybrid electropolymerized films combining polymethylene blue (PMB) and gold nanoparticles (AuNPs) were prepared on screen-printed carbon electrodes (SPCEs) to investigate the electrochemical control of Raman response in redox-active hybrid interfaces. The resulting PMB@AuNP films provide a conductive and electroactive matrix that enables the coupling of electrochemical modulation with Raman spectroscopic readout. The electrochemical behavior of PMB@AuNP/SPCE was dependent on the applied potential, with negative potentials promoting changes in the optical response of methylene blue, evidenced by modulation of the background fluorescence associated with the reduction process. Film composition was systematically evaluated using oppositely charged redox probes, revealing that a PMB:AuNP ratio of 2:1 offers enhanced charge transport and balanced electrochemical response. Under these optimized conditions, the platform was applied to the electrochemical detection of paraquat in aqueous solution, achieving a limit of detection of 4.8 × 10^–7^ mol L^–1^. Spectroelectrochemical measurements further demonstrated that the Raman response is sensitive to the excitation wavelength, with enhanced signals observed under resonance conditions. Overall, these results demonstrate that electropolymerized PMB@AuNP films on SPCEs enable electrochemically controlled Raman response, highlighting their potential as integrated spectroelectrochemical interfaces for sensing applications.

## Graphical abstract

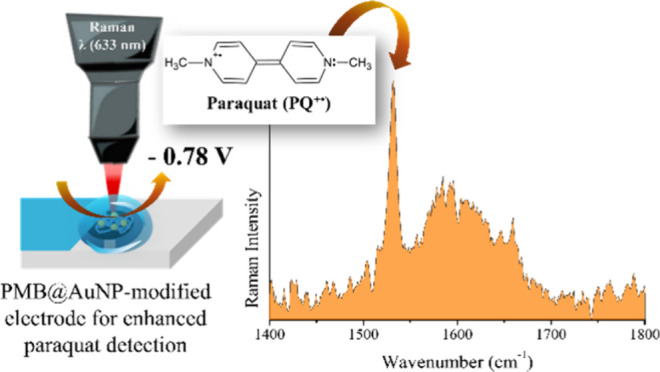

## Introduction

In advanced materials science, developing mixed electropolymerized films, particularly those combining polymethylene blue (PMB) and gold nanoparticles (AuNPs), represents an effective approach for tailoring electroactive and optically responsive interfaces. Polymethylene blue, a conductive polymer, is renowned for its electrochemical stability, redox activity, and versatility in forming thin films.^1^ Gold nanoparticles, on the other hand, offer electrical conductivity, biocompatibility, and unique optical properties due to surface plasmon resonance.^2^ The synergy between these materials enables the design of functional interfaces with tunable electronic and optical characteristics, making them highly attractive for next-generation sensor technologies and electrochemical devices.

Combining optical and electrochemical properties enables real-time monitoring of redox processes in spectroelectrochemical applications. This is essential in areas such as environmental monitoring,^3^ biochemical sensing,^4^ and energy storage devices.^5^ Enhanced interface properties lead to improved signal clarity, faster response times, and greater sensitivity, thereby expanding the capabilities and applications of these innovative materials. However, despite their promising attributes, the controlled synthesis and deposition of such hybrid films remain challenging, particularly when both electrochemical performance and optical response must be optimized within the same electrodeposited film, requiring precise optimization of parameters such as polymerization conditions, nanoparticle loading, and film architecture.

Several studies have demonstrated the potential of films of electropolymerized MB combined with AuNPs in electrochemical applications. For instance, a modified electrode incorporating PMB and AuNPs was successfully employed for the voltammetric detection of serotonin, where the enhanced conductivity and increased active surface area led to significant improvements in sensitivity.^6^ Another study reported the use of PMB/AuNP-modified graphite electrodes for nitrite detection, demonstrating a high electrochemical response compared to unmodified electrodes.^7^ Moreover, PMB-based platforms have been explored for label-free acrylamide detection, further showcasing their versatility in electrochemical sensing applications.^8^ However, despite these advances, the integration of electrochemical techniques with spectroscopic analysis using the spectroelectrochemical approach is largely unexplored, offering a promising avenue for gaining deeper insights into redox mechanisms and improving sensor selectivity.

Understanding the fundamental interactions between PMB and AuNPs at the molecular level is crucial for tailoring their performance in specific applications. Advances in characterization techniques, including spectroscopic and electrochemical analyses, have provided more profound insights into charge transfer mechanisms and film stability. By addressing these challenges, the development of mixed polymer with nanomaterial films can pave the way for breakthroughs in electrochemical sensing, catalysis, and bioelectronics, thereby reinforcing their potential as key materials in future technological innovations. However, most of these studies focus primarily on electrochemical sensing, with limited exploration of spectroelectrochemical modulation as an analytical tool.

Paraquat (1,1′-dimethyl-4,4′-bipyridyl dichloride, PQ), a nonselective contact herbicide widely used in agricultural practices due to its high efficiency and low cost,^9,10^ emerges as an analyte to evaluate the performance of such hybrid interfaces. Although it acts rapidly and is cost-effective, its high solubility, extreme toxicity, and environmental persistence have led to serious contamination of soil and aquatic ecosystems, as well as significant health risks.^11,12^ Because of these hazards, PQ has been classified as a hazardous chemical by the U.S. Environmental Protection Agency (US-EPA), and its use has been restricted in several regions, including the European Union and numerous Latin American countries.^13^

Analytical techniques such as spectrophotometry, chromatographic, spectrometric methods, and Raman-based approaches provide high accuracy for PQ quantification.^9,14,15^ However, these methods are often complex, are time-consuming, and require costly instrumentation, limiting their application in field monitoring.^10,15^ In contrast, electrochemical sensors represent a simple, rapid, sensitive, and portable alternative.

From an electrochemical perspective, PQ exhibits well-defined and reversible redox behavior involving two successive one-electron reductions. These redox transitions produce distinct analytical signals, making PQ an ideal electroactive probe to assess electron transfer efficiency and sensitivity in modified electrode systems.^15,16^ Additionally, diquat (1,1′-ethylene-2,2′-bipyridyl dibromide, DQ), a structural analogue of PQ, displays a similar electrochemical profile to PQ, as both are bipyridinium herbicides capable of undergoing comparable redox transformations.^17^ This resemblance provides a valuable opportunity to employ spectroelectrochemical techniques to examine subtle differences in adsorption, orientation, and charge transfer processes between the two analytes and the electrode interface.^12,15^ Therefore, the study of PQ on nanostructured hybrid films provides a suitable electroactive model to probe charge transfer and adsorption phenomena at hybrid electrode interfaces.

In this work, the interfacial interaction of a hybrid film composed of PMB and AuNPs (PMB@AuNP) on screen-printed carbon electrodes (SPCE) was explored through its electrochemical behavior, and the modified electrode was evaluated as both an electrochemical and Raman spectroelectrochemical sensor platform, using paraquat (PQ) as a target molecule.

## 2 Materials and Methods

### 2.1 Reagents and Solutions

Methylene blue monomer (Synth), tetrachloride auric (III) acid, HAuCl_4_ (Aldrich), potassium chloride (KCl), potassium hexacyanoferrate­(II) (Sigma-Aldrich), potassium hexacyanoferrate­(III) (Sigma-Aldrich), hexaammineruthenium­(II) chloride (Sigma-Aldrich), and paraquat (Sigma-Aldrich, analytical grade) were used without further purification. The methylene blue (MB) solution (2 mmol/L) and HAuCl_4_ solution (0.5 mmol/L) were prepared separately in the presence of 0.1 mol/L KCl as supporting electrolyte. A 0.01 mol/L paraquat (PQ) solution was used as the stock solution. All aqueous solutions were prepared using deionized water (3.0 μS/cm). The electrochemical interfacial properties and spectroelectrochemical analysis were evaluated for the film electrodeposited on screen-printed carbon electrodes (SPCE, Dropsens 110) that have a carbon auxiliary electrode and a Ag reference electrode.

### 2.2 Electrodeposition of Poly­(methylene blue) and Gold Nanoparticles

The electrodepositions performed were as follows: (i) PMB formation on SPCE, then the electrodeposition of gold nanoparticles (AuNPs), by one of the methods described in (ii) and (iii), summarized in the scheme shown in Figure 1A.

(i) Electrodeposition of PMB: 5, 10, and 20 potential cycles in the range of −0.7 to 1.2 V at 100 mV/s using 2 mmol/L MB solution. The PMB modified electrode surface (PMB/SPCE) was washed with deionized water and dried at room temperature.
(ii) Covering the PMB with AuNPs, also obtained by electrodeposition, to give AuNP/PMB/SPCE. The PMB-modified electrode (with 20 potential cycles) was immersed in HAuCl_4_ solution (0.5 mmol/L), and five potential cycles were applied in the potential range of −1.0 to 0.2 V at 100 mV/s. The electrode surface was again washed with deionized water and dried at room temperature.
(iii) Hybrid electrodeposition, PMB@AuNP to give PMB@AuNP/SPCE. The electrodeposition was performed using a mixture of MB (2 mmol/L) and HAuCl_4_ (0.5 mmol/L) solution in molar ratios of 10:1, 4:1, and 2:1 of MB:HAuCl_4_. The PMB@AuNP film was electrodeposited by applying 20 potential cycles from −1.0 to 0.2 V at a scan rate of 100 mV/s.

**Figure 1:**
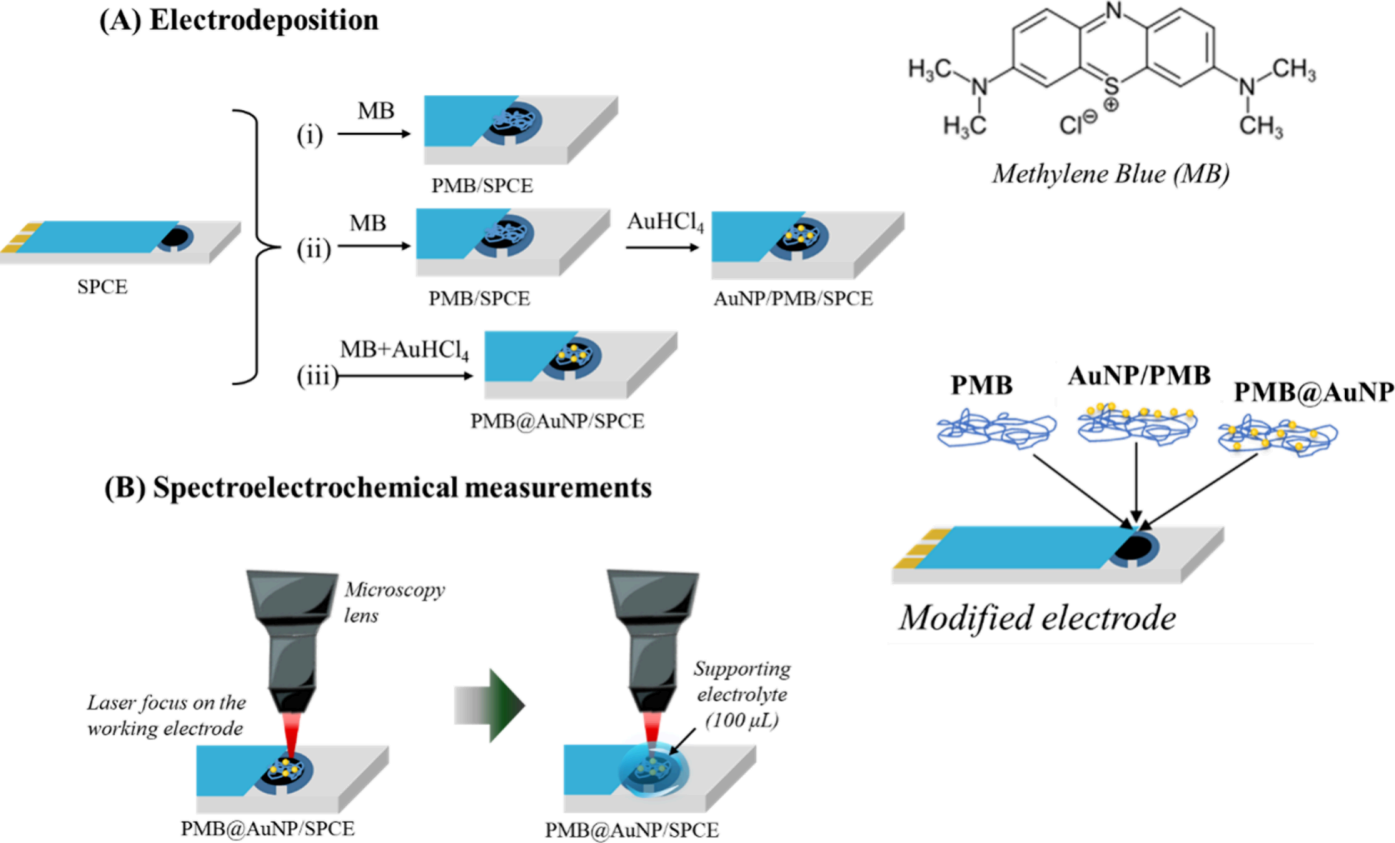
Schematic representation of (A) electrodeposition steps for fabrication of PMB, PMB/AuNP, and PMB@AuNP-modified SPCE. (B) Representation of the setup for electrochemical measurements using electrochemical and Raman spectroscopy techniques. The methylene blue structure and film nomenclature are also represented.

Evaluation of the electrodeposition mechanism by Raman spectroscopy was performed at a fixed applied potential of −0.187 V (cathodic process). The microscope lens was focused on the working electrode before adding electrolyte solution. Then, 100 μL of the electrolyte solution containing the active molecules (MB or a mixture of MB and HAuCl_4_) was dropped onto the working electrode, and measurements were carried out by collecting a total of 20 Raman spectra (one spectrum per minute).

### 2.3 Instrumentation

Electrodeposition, cyclic voltammetry, and electrochemical impedance spectroscopy (EIS) measurements were performed using a portable potentiostat, Dropsens model STAT400S. The measurements were performed using 100 μL of supporting electrolyte (0.1 mol/L KCl) and redox probes [Fe­(CN)_6_]^3–^/[Fe­(CN)_6_]^4–^ and [Ru­(NH_3_)_6_]^2+^. Cyclic voltammetry was performed between −1.0 and +1.0 V at 50 mV/s for the analysis of the KCl solution, while for the probe solution, it was performed between −0.2 and 0.7 V at 50 mV/s. In [Fe­(CN)_6_]^3–^/[Fe­(CN)_6_]^4–^ solution, a study of the influence of scan rate from 5 to 200 mV/s was performed and the active area was also calculated, using the Randles–Sevick equation:

$$I_{p}=2.69\times 10^{5}AD^{1/2}n^{3/2}v^{1/2}C^{*}$$ 1

where *I*_p_ is the peak current, *A* is the effective surface area (cm^2^), *D* is the diffusion coefficient of [Fe­(CN)_6_]^3–^ species (7.6 × 10^–6^ cm^2^/s^18^), *n* is the number of electrons transferred (*n* = 1), *v* is the scan rate, and C* is the bulk concentration of [Fe­(CN)_6_]^3–^ (2.0 × 10^–6^ mol/cm^3^). The effective surface area was estimated from the slope of the plot *I*_p_ = *f* (*v* ^1/2^).

EIS measurements were made for modified electrodes at open-circuit potential (OCP) in the frequency range of 65 kHz to 0.1 Hz, with 10 frequency steps per decade, and a sinusoidal voltage perturbation of 10 mV rms amplitude. Impedance spectra were analyzed by fitting them to equivalent electrical circuits using DropView 8400 software.

Raman spectra were collected from the surface of the PMB, PMB/AuNP, and PMB@AuNP film-modified SPCE and compared with the spectrum of MB powder. Characterization was performed using a Renishaw micro-Raman spectrophotometer, model inVia, coupled to a Leica microscope (objective lens 50×). The spectra were acquired using the laser line at 633 nm, grating of 1800 L/mm, with 10 s acquisition time and with a laser power of 2.5 mW at the sample (measured at the objective lens output). The same equipment was used in the spectroelectrochemical measurements.

Surface morphology was examined using scanning electron microscopy (SEM) with a ZEISS scanning electron microscope model EVO LS15 at an accelerating voltage of 20.0 kV. The images were collected using a backscattering secondary electron (BSE) detector without a gold surface coating. The SEM images were acquired for PMB/SPCE and PMB@AuNP/SPCE at a 2:1 molar ratio.

### 2.4 Voltammetric and Spectroelectrochemical Analyses of Paraquat

The stability, reproducibility, and repeatability of the PMB@AuNP/SPCE were evaluated by cyclic voltammetry in the presence of 0.1 mmol/L of PQ with 0.1 mol/L KCl as supporting electrolyte, cycling from −1.0 to +1.0 V at 50 mV/s. The PMB@AuNP/SPCE was also applied as an electrochemical sensor to evaluate its analytical behavior from 4.0 × 10^–8^ to 4.0 × 10^–6^ mol/L using cyclic voltammetry.

Recovery analysis was performed using river water samples. The water was collected from the Paraná River in Ilha Solteira, São Paulo State, Brazil (gps coordinates −20.386129, −51.345586), following ISO 5667 guidelines, having a pH of 5.0 and a conductivity of 91.9 μS cm^–1^. The measurements were carried out 1 h after sample collection. A 0.1 mol/L KCl solution was prepared using the river water without any dilution. Small volumes of a PQ standard solution were spiked into the samples for performed recovery measurements. In this case, 10^–2^ mol/L PQ standard solution was used to obtain final concentrations of 1.0, 1.6, 2.0, and 4.0 μmol/L in the samples for voltammetric measurements.

In the presence of PQ solution, the Raman spectra were collected after an equilibration time (60 s) at an applied potential of −0.78 V (identified as the cathodic peak of PQ reduction). Raman spectra were also collected without an applied potential as a reference (OCP mode) as comparison. The spectroelectrochemical measurements were carried out using the 633 and 785 nm laser lines for Raman spectral acquisition.

### 2.5 Spectroelectrochemical Characterization

The spectroelectrochemical measurements were performed by coupling the portable potentiostat to the micro-Raman microscope, in order to examine the response of the PMB and PMB@AuNP-modified electrode surfaces, as represented in Figure 1B. The laser was focused on the working electrode surface, and 100 μL of the solution (supporting electrolyte, monomer, or probe solution) was dropped on the surface, covering the three electrodes (reference, working, and counter electrodes). The Raman spectra were collected during the PMB and PMB@AuNP electrodeposition. Additionally, after electrodeposition, the electrochemical behavior of the PMB@AuNP film-modified SPCE was evaluated by cyclic voltammetry in supporting electrolyte solution, and Raman spectra were collected during both the anodic and cathodic processes. In this case, the line mapping function was applied to obtain the Raman spectra. The Raman measurements were performed using a laser at 633 and 785 nm.

## 3 Results and Discussion

### 3.1 Formation and Voltammetric Behavior of Films by Potential Cycling

The formation of PMB polymer by electrodeposition can be ascribed to the direct interaction of ring-to-ring coupling through the amino group in the carbon atom in the ortho-position (C–N coupling) or through nitrogen bridges.^1,8,19^ The electrodeposition mechanism proposed for polymer formation through C–N coupling starts with the formation of cation radicals during monomer oxidation at a potential higher than +0.80 V.^20^ This polymerization process can be influenced by experimental conditions such as scan rate, number of scans, monomer concentration, pH, or electrolyte.^21^

During the electrodeposition of PMB, an increase in both anodic and cathodic processes was observed with increasing number of potential cycles (Figure 2A and Figure S1A–C), as expected. Specifically, the polymer redox process exhibited higher current values after 20 potential cycles than after 5 and 10, since the amount of polymer formed increases with the number of cycles. The electrodeposition process of the MB film (Figure 2A) displayed a redox pair at −0.224 and −0.317 V, attributed to the oxidation and reduction processes of the MB monomer. After the second potential cycle, a new redox process was observed with a maximum anodic current at 0.042 V, attributed to the formation and progressive growth of the PMB film, which shifted to more positive potentials as the number of cycles increased (to 0.166 V after 20 cycles). The observed values are consistent with those reported by Schlereth and Karyakin.^22^ The increasing number of potential cycles during electrodeposition contributed to an increase in charge transfer (Figure 2B) in the [Fe­(CN)_6_]^3–^/[Fe­(CN)_6_]^4–^ redox probe and an increase in the effective surface area, as calculated using the Randles–Sevcik equation (Figure 2C,D and Figure S2D–F). The electroactive surface areas were 0.140, 0.154, and 0.208 cm^2^ for 5, 10, and 20 potential cycles, respectively, higher than the geometric area of the carbon electrode (0.110 cm^2^) and increasing with the number of potential cycles. Therefore, the PMB film electrodeposited using 20 cycles was selected as the reference for subsequent modification with AuNPs.

**Figure 2:**
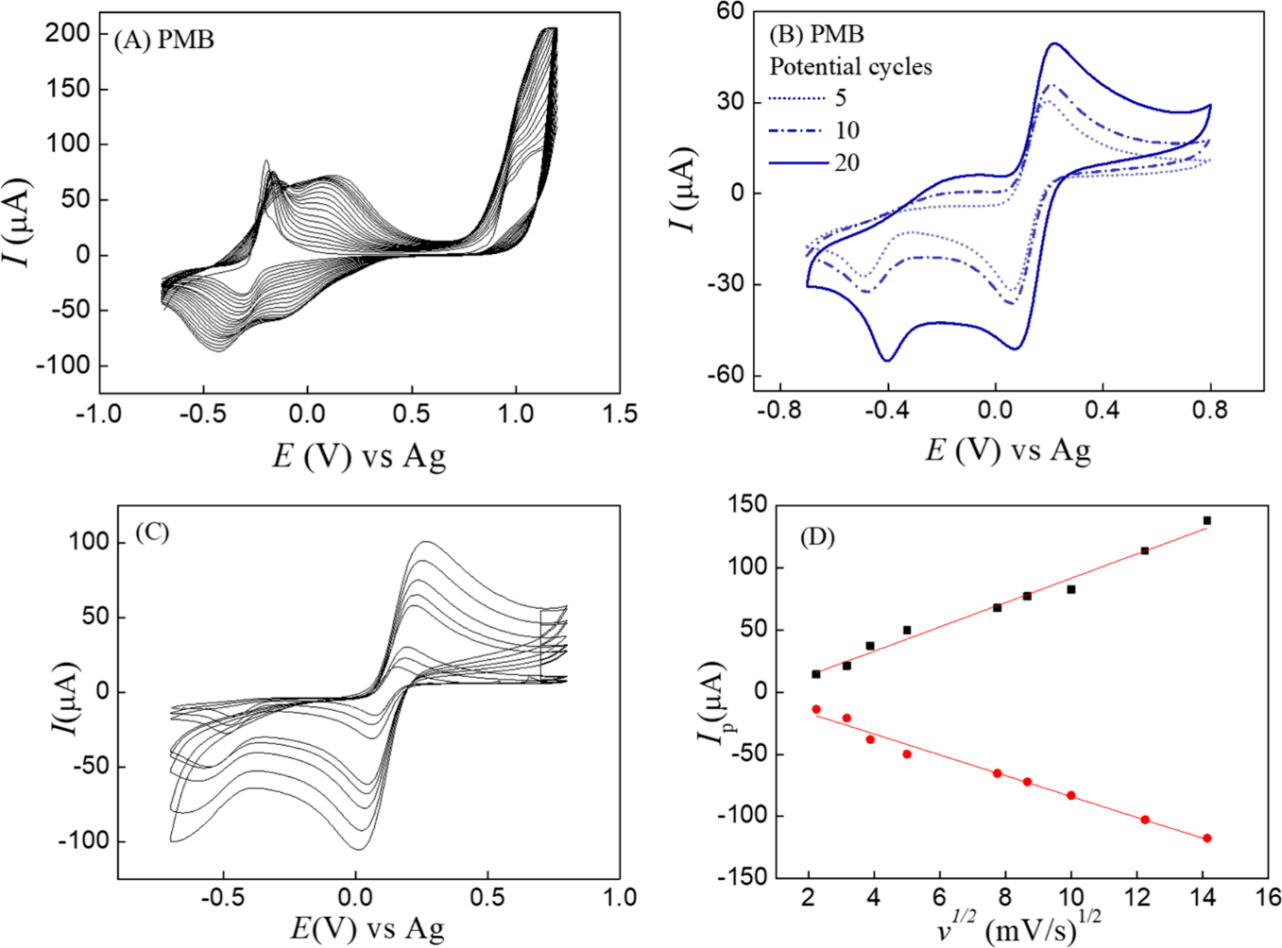
(A) Cyclic voltammograms obtained during the electrodeposition of PMB films for 20 potential cycles at 50 mV/s. (B) Cyclic voltammograms in 2 mmol/L [Fe­(CN)_6_]^3–^/[Fe­(CN)_6_]^4–^ solution for the PMB films electrodeposited for 5, 10, and 20 potential cycles. (C) Cyclic voltammograms for scan rates from 5 to 200 mV/s in 2 mmol/L [Fe­(CN)_6_]^3–^/[Fe­(CN)_6_]^4–^ solution. (D) Peak current vs square root of scan rate for the PMB film-modified SPCE obtained with 20 potential cycles.

### 3.2 Incorporation of AuNP in PMB Films

The electrodeposition of PMB films was also evaluated for two different architectures, with AuNP as a coating layer (PMB/AuNP) and with AuNP incorporation into the polymeric matrix to produce a nanocomposite (PMB@AuNP). The incorporation of nanoparticles into PMB films is generally performed using chemically synthesized AuNPs. Cruz-Pacheco et al.^23^ performed the electrodeposition of PMB using a colloid of sodium citrate-coated AuNP, where the cyclic potential scanning promotes the voltammetric polymerization of MB with insertion of nanoparticles into the polymer matrix.^23^ Abad-Gil and Brett^20^ evaluated the polymerization of MB on a glassy carbon electrode previously modified by the adsorption of AuNP and chitosan. The electrodeposition was carried out in a eutectic solvent, and the results indicate that the presence of AuNP on the electrode surface enhances the stability of the polymer film and improves the sensing performance.

In this study, AuNPs were investigated in two configurations: as a surface coating layer and as a nanocomposite formed by in situ growth during a potential cycling process. This procedure avoids chemical synthesis, and adsorption and insertion occur directly in the polymer matrix during structure formation.

The AuNP electrodeposited onto the PMB surface as a coating layer was performed by applying five potential cycles (Figure 3A). A pronounced peak in the range from −0.3 to −0.6 V can be interpreted as the adsorption of the gold precursor onto the electrode surface^24^ or as the nucleation of gold seeds.^25^ At more negative potentials (−0.6 V), the nucleation and growth of gold particles occur concurrently.^25^ The coating of the PMB surface with AuNP resulted in an increase of the electroactive area to 0.214 cm^2^, which is higher than that of PMB with 20 potential cycles (0.208 cm^2^). This increase in electroactive area can be attributed to the nanoparticles at the electrode surface, which exhibit spherical shapes. Koyun and Sahin^7^ described the electrodeposition of AuNP at fixed potential onto the PMB surface. The authors demonstrated the formation of gold nanoflowers on the electrode surface, which contributed to increased sensitivity by enhancing the conductivity and surface area.

**Figure 3:**
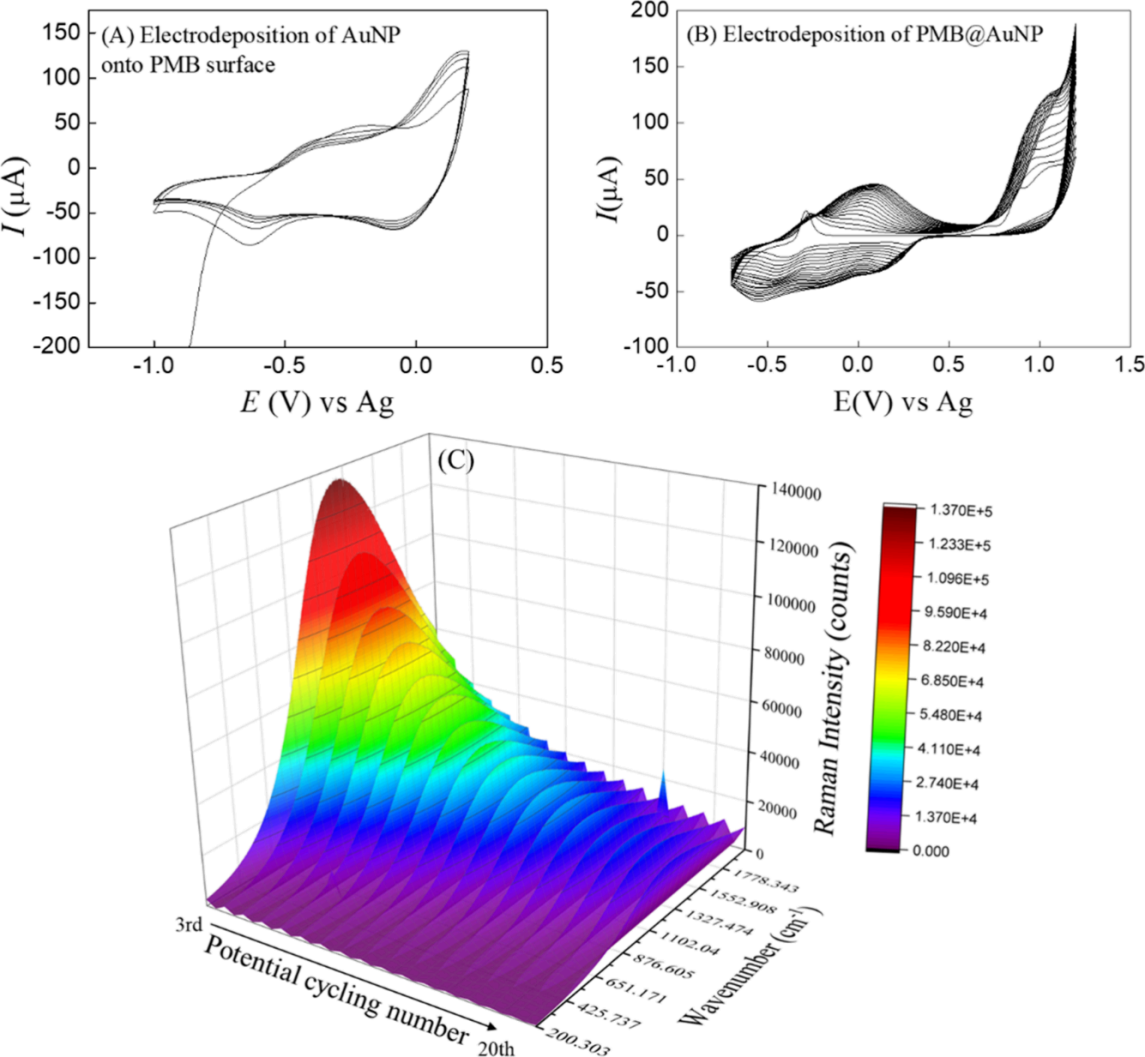
Cyclic voltammogram obtained during (A) the electrodeposition of AuNP onto the PMB film surface and (B) the electrodeposition of the nanocomposite PMB@AuNP (ratio 2:1). (C) Raman spectra recorded during the electrodeposition of the nanocomposite PMB@AuNP (ratio 2:1) by fixed potential electrodeposition at −0.187 V.

In the case of nanocomposite formation (PMB@AuNP), the cyclic voltammogram of PMB overlapped with the characteristic signals of AuNP deposition (Figure 3B). However, in the first potential cycle (Figure S3), a new anodic peak at 0.888 V was observed, which can be assigned to the adsorption of MB monomer onto the previously formed AuNP. The electroactive area increased with higher concentrations of Au­(III) ions during electrodeposition, reaching values of 0.208 cm^2^ (10:1), 0.260 cm^2^ (4:1), and 0.275 cm^2^ (2:1). This highlights the influence of the Au content on the formation of the nanostructured film, and an increase of deposited material, with more nanoparticles leading to greater contact area.

The PMB@AuNP nanocomposite electrodeposition was also monitored *in situ* by spectroelectrochemical measurements using a Raman excitation source at laser line 633 nm. The PMB@AuNP/SPCE was obtained using a potentiostat coupled to a Raman spectrophotometer, and one spectrum was collected per minute over 20 min electrodeposition at −0.187 V. Under these conditions, the recorded spectra showed a broad fluorescence background, with no discernible Raman vibrational bands (Figure 3C). This is consistent with the well-known fluorescence background of MB when excited at 633 nm, particularly in solution.^26^

However, a decrease in the fluorescence background was observed with increasing electrodeposition time. This behavior can be attributed to fluorescence quenching processes occurring at the electrode–electrolyte interface. Such quenching is likely related to the electropolymerization of MB, which reduces the radiative relaxation pathways of the excited species and may also be influenced by interfacial effects arising from the presence of gold nanoparticles, including nonradiative decay channels promoted by plasmonic interactions. While these in situ measurements do not provide direct vibrational or mechanistic information regarding the polymerization pathway, they offer indirect optical evidence of interfacial evolution during film growth.

The formation of the polymer film was confirmed through Raman spectra collected at a dry working electrode surface, after electrodeposition (Figure 4). The PMB film showed the main bands observed in the MB monomer (powder), which are highlighted in Figure 4A. The electrodeposited films displayed a new band at 1565 cm^–1^, attributed to the −N­(CH_3_)_2_ stretching of the aromatic rings in MB,^27^ confirming the formation of the polymer on the electrode surface without disruption of the monomeric structure. Thus, our results indicate that the electrochemical polymerization of MB proceeds either through direct ring-to-ring coupling or via nitrogen bridges.^8^

**Figure 4:**
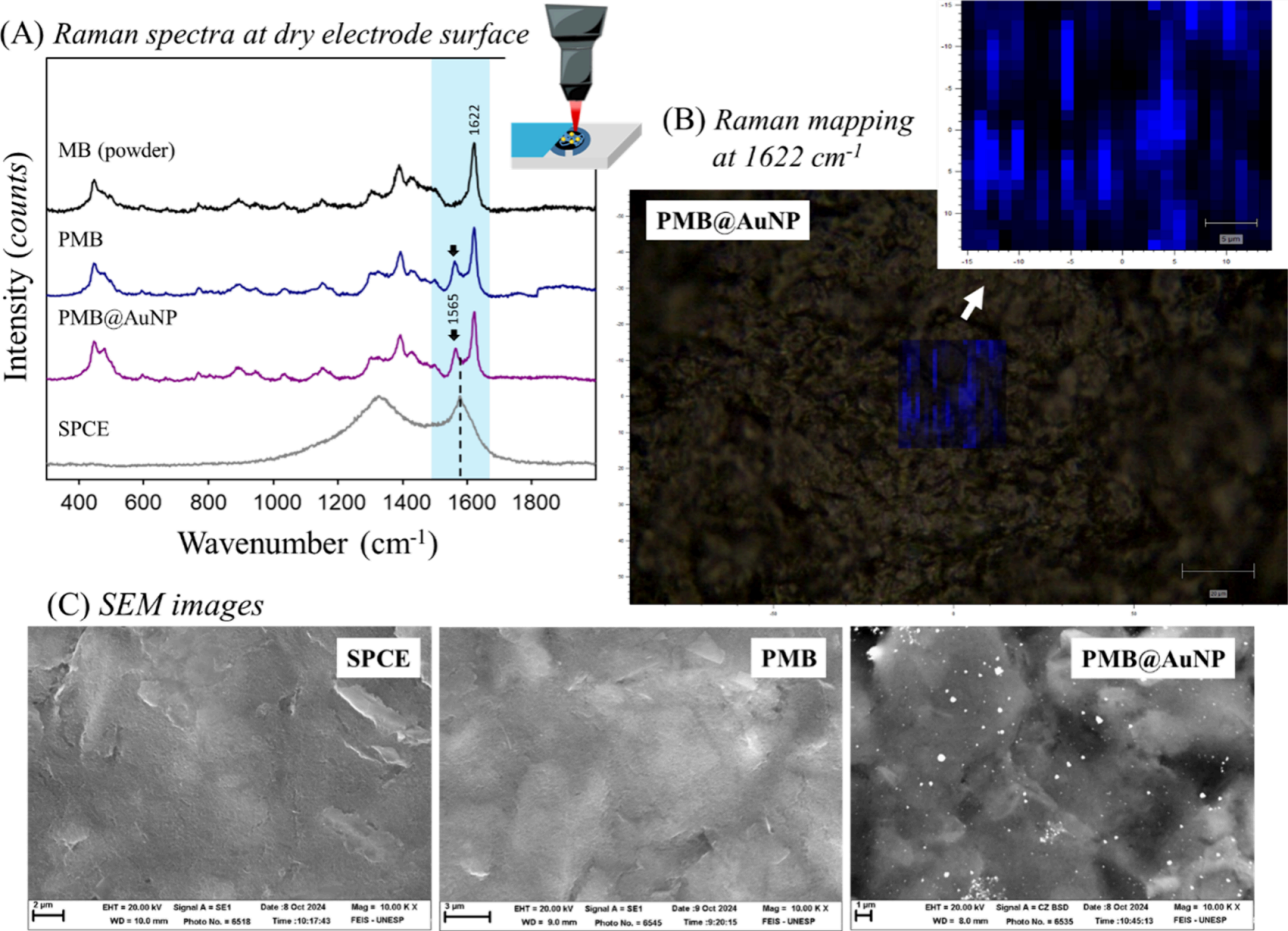
(A) Raman spectra collected on the surface of the working electrode modified with PMB (20 potential cycles), the PMB@AuNP nanocomposite, and the unmodified SPCE. The Raman spectrum of MB powder was also obtained as a reference. (B) Raman mapping of a 25 × 25 μm area for the band at 1622 cm^–1^ corresponding to the PMB@AuNP nanocomposite. (C) SEM images of the unmodified SPCE, and the electrodes modified with PMB (20 potential cycles) and the PMB@AuNP nanocomposite (molar ratio 2:1). SEM images with magnification of 10.000×*.* Scale bars of 2, 3, and 1 μm.

The Raman spectra for the nanocomposite PMB@AuNP-modified SPCE exhibited the same band positions and relative intensity profile as those observed for PMB-modified SPCE. This indicates that the presence of the Au­(III) ion precursor and the resulting Au nanoparticles did not alter the chemical structure of MB during the electropolymerization process. The observation of only the conventional Raman effect can be associated with limited plasmonic coupling and/or insufficient hot-spot formation, as well as a nonoptimal overlap between the excitation wavelength and the localized surface plasmon resonance of the Au nanoparticles. This behavior depends not only on the mere presence of AuNPs but also on nanoparticle size, surface coverage, interparticle gaps, and plasmonic coupling conditions. High enhancement factors are typically associated with “hot spots” formed at nanometer-scale junctions between closely spaced or aggregated nanoparticles, which was not observed in our results. These results also agree with the cyclic voltammograms, which showed no significant changes in the electrodeposition profile, suggesting that PMB polymerization is not significantly altered by the concurrent formation of AuNPs.

Raman mapping using the band signal at 1622 cm^–1^ showed that the electrodeposition of PMB follows the SPCE morphology (Figure 4B). Indeed, the SEM images showed no significant difference between the unmodified SPCE and SPCE modified with PMB (Figure 4C). However, the formation of AuNPs as clusters over the electrode surface was confirmed (Figure 4C). The AuNP clusters showed a mean size distribution of 228 ± 134 nm. The size histogram and complementary SEM images are shown in Figure S4.

### 3.3 Electrochemical Interface and the Charge Transfer

To clarify the role of surface charge and film morphology, the interfacial properties were evaluated using oppositely charged redox probes. The charge transfer properties of the nanocomposites were evaluated using negatively and positively charged redox probes, [Fe­(CN)_6_]^3–^/[Fe­(CN)_6_]^4–^ and [Ru­(NH_3_)_6_]^2+^ (Figure 5), to understand the influence of surface charge and interfacial electrostatic interactions on the electron transfer kinetics at the modified electrode interface, as reflected by changes in Δ*E*_p_, *I*_pa_/*I*_pc_ ratios, and charge transfer resistance (*R*_ct_). For both redox probes investigated, the electrochemical signals were slightly more reversible for the SPCE-modified electrodes than for the unmodified electrode, accompanied by a small decrease in the electroactive area of the modified electrode compared to the unmodified SPCE (0.232 cm^2^). Comparing the PMB films with the nanocomposite PMB@AuNP as modifiers, the incorporation of AuNP in the polymer matrix leads to favorable electrostatic interactions with the redox probes with an increase of electroactive area to 0.275 cm^2^ (at 2:1 ratio). Kayun and Sahin^7^ reported that for PMB films with AuNP on graphite electrodes (derived from pencils), the peak current increases proportionally with the increase in the electroactive area in the presence of the [Fe­(CN)_6_]^3–^/[Fe­(CN)_6_]^4–^ redox probe.^7^ However, in our case, the nanocomposite PMB@AuNP electrodeposited with different MB:Au molar ratios (Figure 5B) showed no significant changes in peak current and in the difference between the anodic and cathodic peak potentials for the [Fe­(CN)_6_]^3–^/[Fe­(CN)_6_]^4–^ redox probe. This behavior can be attributed to the decreased interaction resulting from the repulsion caused by AuNP and the redox probe, both of which are negatively charged. In the case of the two-layer AuNP/PMB films, the external AuNP coating can increase the redox probe repulsion and decrease the hexacyanoferrate peak current, as highlighted in Figure 5A1. For PMB with 5, 10, and 20 potential cycles, there is a proportional increase of peak current (Figure 2B) of the [Fe­(CN)_6_]^3–^/[Fe­(CN)_6_]^4–^ redox probe, indicating a favorable interaction between the positive charge of PMB and the negative redox probe and a constant rate of PMB deposition.

**Figure 5:**
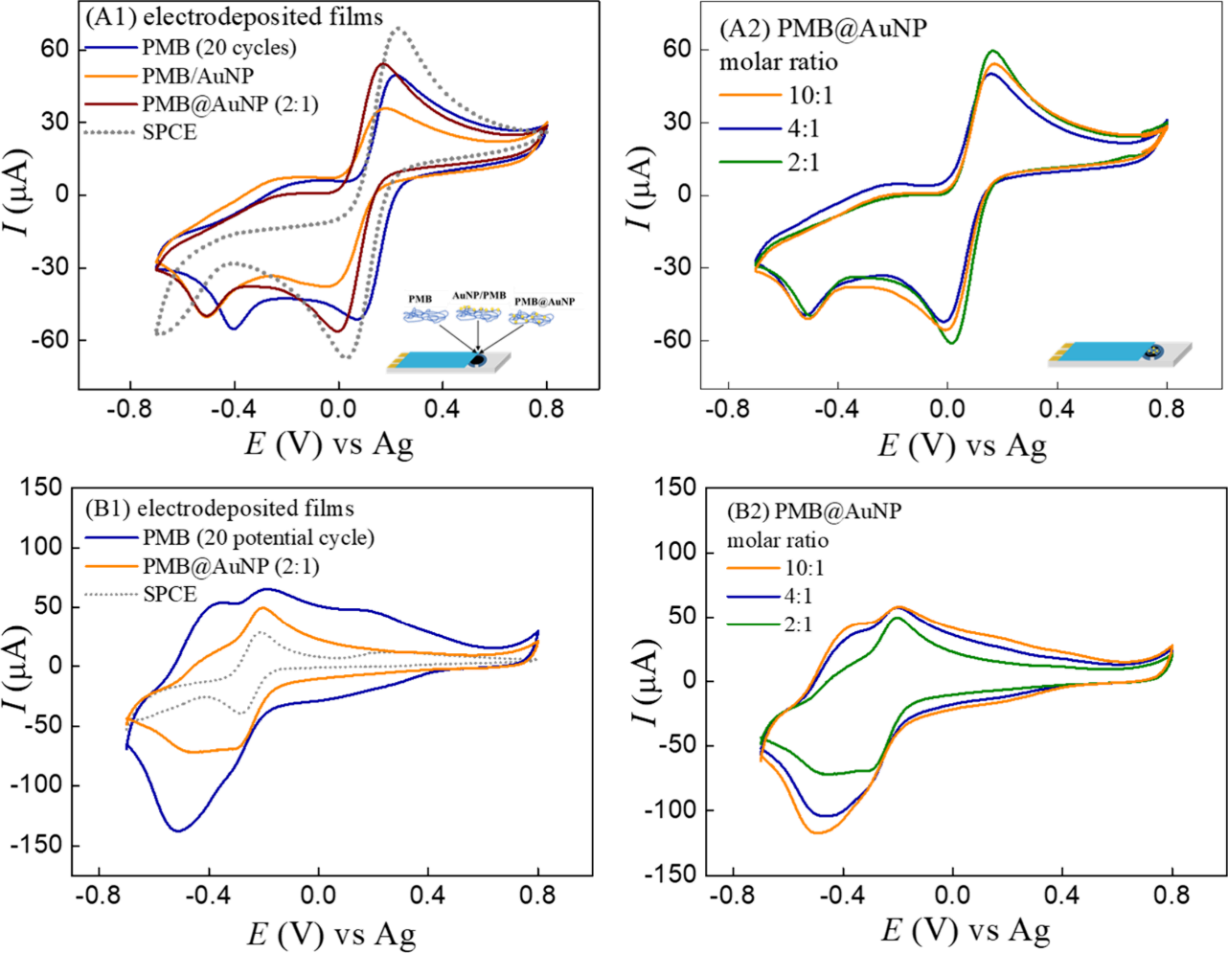
(A) Cyclic voltammograms recorded in the presence of 2 mmol/L [Fe­(CN)_6_]^3–^/[Fe­(CN)_6_]^4–^ at 50 mV/s for (A1) PMB, PMB/AuNP, PMB@AuNP nanocomposite (2:1), and the unmodified SPCE, and (A2) PMB@AuNP nanocomposites prepared with different MB:Au molar ratios (10:1, 4:1, and 2:1). (B) Cyclic voltammograms recorded in the presence of 2 mmol/L [Ru­(NH_3_)_6_]^2+^ at 50 mV/s for (B1) PMB, PMB@AuNP nanocomposite (2:1), and the unmodified SPCE; and (B2) PMB@AuNP nanocomposites prepared with different MB:Au molar ratios (10:1, 4:1, and 2:1).

The preference of the PMB@AuNP nanocomposite for electrostatic interactions with cationic species was reinforced by the cyclic voltammograms recorded with the positively charged [Ru­(NH_3_)_6_]^2+^ redox probes (Figure 5B). The PMB@AuNP nanocomposite exhibited greater redox reversibility than PMB, as evidenced by the lower peak-to-peak separation (Δ*E* = 98 mV) compared to that of the PMB film (Δ*E* = 129 mV), indicating improved charge transfer of cationic species. However, for nanocomposite PMB@AuNP electrodeposited with different molar ratios, no significant changes were observed for 10:1 and 4:1 molar ratios. Additionally, in the presence of the [Ru­(NH_3_)_6_]^2+^ redox probe, a redox couple at −0.378 V (shoulder)/–0.468 V was also observed, which is ascribed to the redox process of the PMB polymer.

The cyclic voltammogram for PMB@AuNP/SPCE, recorded in inert supporting electrolyte (0.1 mol/L KCl), showed two redox couples at −0.180/–0.530 and 0.252/0.165 V, attributed to the redox processes of the polymer and monomer, respectively (Figure S1). However, in the cyclic voltammogram for PMB, the redox process of the monomer is less intense than that observed with the nanocomposite. This behavior suggests that the presence of AuNP in the polymer matrix increases monomer adsorption during electrodeposition. Thus, in the presence of [Ru­(NH_3_)_6_]^2+^, the two electrochemical reactions (PMB and [Ru­(NH_3_)_6_]^2+^) occur simultaneously, resulting in substantial signal amplification of the redox process of PMB. The latter is not observed in the measurements carried out with the negative redox probe.

Charge transfer was also evaluated using EIS in the presence of the redox probes [Fe­(CN)_6_]^3–^/[Fe­(CN)_6_]^4–^ and [Ru­(NH_3_)_6_]^2+^. The Nyquist plot (Figure 6A) for the unmodified SPCE shows a semicircular region at high frequencies ascribed to the electron transfer process, followed by a straight-line part at low frequencies ascribed to the diffusion-limited process.^28^ Fitting was done by a Randles-type equivalent circuit, composed of a cell resistance, *R*_Ω_, in series with a parallel combination of a constant phase element, CPE, and a charge transfer resistance, *R*_ct_, together with a Warburg impedance, *Z*_w_, as shown in Figure 6B. After modification, changes were observed in both the semicircular region at high frequencies and the straight-line part at low frequencies, indicating that the polymer film structure influences the interfacial properties. Moreover, the difference in the impedance profiles for negative and positive redox probes indicates that the ionic species in solution play an important role in the charge transfer resistance and enhance the influence of mass transport phenomena.

**Figure 6:**
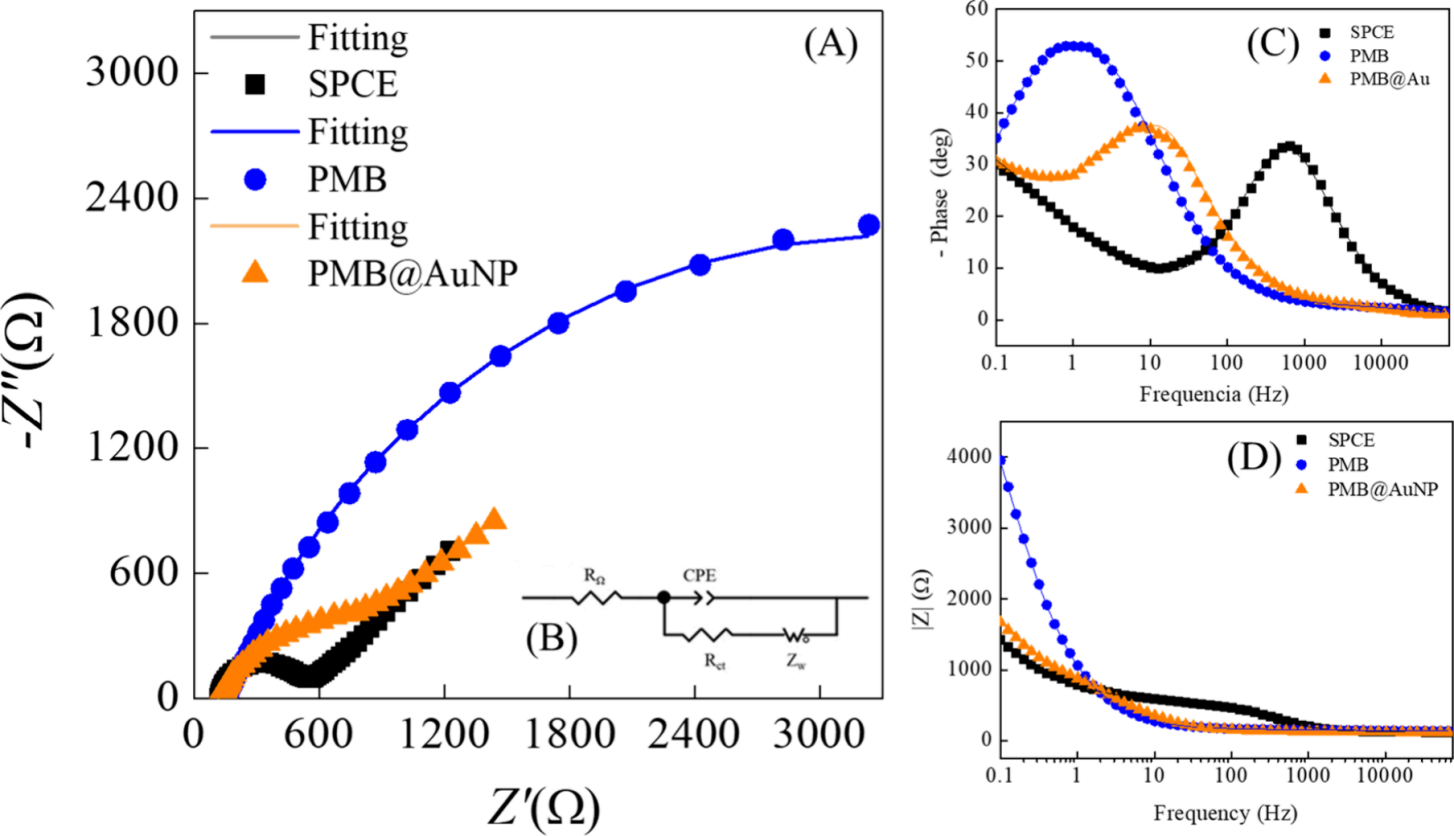
(A) Complex plane impedance, (B) equivalent circuit, (C) impedance Bode plot, and (D) phase degree impedance spectra obtained for SPCE unmodified and modified with PMB and PMB@AuNP. EIS measurements carried out at OCP in 2 mmol/L [Fe­(CN)_6_]^3–^/[Fe­(CN)_6_]^4–^ redox probe in 0.1 M KCl solution.

With the [Fe­(CN)_6_]^3–^/[Fe­(CN)_6_]^4–^ redox probe, the modification with PMB promotes an increase of interfacial capacitance, *Z*w being negligible, suggesting fast ion diffusion.^29^ This behavior is further supported by the shift of the phase angle and the increase in impedance at low frequencies (Figure 6C,D). The PMB@AuNP/SPCE showed an intermediate behavior between the PMB-modified and unmodified electrodes. The values of the elements of the equivalent circuit calculated from fitting the impedance spectra are presented in Table 1. Insertion of AuNP plays an important role in ion migration to the double-layer capacitance, increasing with the molar ratio of AuNP (Figure S5). From the complex plane (Nyquist) plots (Figure S5), it was observed that the films corresponding to the 2:1 molar ratio system exhibited lower charge transfer resistance compared to the other molar ratios (10:1 and 4:1), corresponding to greater electron transfer. These findings suggest that the combination of PMB film and AuNP in this ratio was the most favorable for forming films with the best properties, of those tested, for electrochemical sensor applications.

**Table 1:** Parameter Values Obtained from Fitting the Impedance Spectra Recorded at OCP to the Equivalent Circuits, Data from Fig. 6

**electrode**	*R*_s_ (Ω)	*R*_ct_ (Ω)	CPE (μF s^α–1^)	α	*Z*_W_ (Ω)
*Measurements carried out with [Fe(CN)*_*6*_ *]* ^*3–*^ */[Fe(CN)*_*6*_ *]* ^*4–*^					
**SPCE**	119	384.5	3.7	0.88	0.0012
**PMB/SPCE**	166	3545	321.7	0.70	
**PMB@AuNP/SPCE**	118	452	154	0.89	0.0010
*Measurements carried out with [Ru(NH*_*3*_ *)_6_]* ^*2+*^					
**SPCE**	129		6.2	0.90	1.43 × 10^–6^
**PMB/SPCE**	148		461	0.81	8.75 × 10^–4^
**PMB@AuNP/SPCE**	116		110	0.82	4.70 × 10^–4^

In the presence of the [Ru­(NH_3_)_6_]^2+^ redox probe, the unmodified SPCE showed an almost linear spectrum tilted with respect to the imaginary impedance axis with depressed semicircles in the Nyquist plot, indicating a blocking electrode behavior.^29^ Thus, the equivalent circuit for fitting includes only the CPE and *Z*w components (Figure S6), indicating a low affinity of the electrode surface to cationic species, less favorable to charge transfer. Modification with PMB promotes an increase in charge transfer in the presence of cationic species through favorable electrostatic interactions. This observation is consistent with the cyclic voltammetry results, where the PMB films exhibit higher faradaic currents due to the [Ru­(NH_3_)_6_]^2+^ redox probe. Incorporating AuNPs leads to an intermediate behavior, since the gold nanoparticles modify the active surface properties, resulting in a response more similar to that of the unmodified SPCE.

### 3.4 Cyclic Voltammetry of Paraquat at PMB@AuNP/SPCE

The PMB@AuNP/SPCE was evaluated by cyclic voltammetry in the presence of paraquat (PQ). PQ showed two redox processes ascribed to the reduction of cation species (PQ^+^) to the radical cation (PQ^+^·) followed by reduction to the neutral species (PQ^0^), as follows:

$${\mathrm{PQ}}^{2+}+e^{-}⇌{\mathrm{PQ}}^{\cdot +}$$ 2

$${\mathrm{PQ}}^{\cdot +}+e^{-}⇌{\mathrm{PQ}}^{0}$$ 3

For PMB@AuNP/SPCE, the cathodic process corresponding to the formation of PQ^·+^ overlaps with the cathodic response of monomeric MB species present in the nanocomposite (Figure 7A). Increasing the PQ concentration promotes only a shift toward a more positive potential (Figure 7B). The cathodic process at −0.78 V, ascribed to the reduction of the PQ radical species, showed an increase in current with increasing PQ concentration, which was used for quantitative analysis (Figure 7C). The cathodic current at −0.78 V exhibited a linear increase in the range of 0.6 to 4.0 μmol/L, as shown in Figure 7B–D, with a limit of detection (LOD) of 4.8 × 10^–7^ mol/L (3×SD/Slope, where SD is the standard deviation of the blank and slope from the calibration curve). The linear regression equation is −*I*_p_(μA)= 27.122 + 3.528 [PQ] (μmol/L), with *R* ^2^ = 0.996, *n* = 7, showing a sensitivity of 3.5 μA/μmol/L. The reproducibility of the sensor was evaluated using three independently modified electrodes, while repeatability was assessed through 25 consecutive potential cycles. The relative standard deviation (RSD) values were 2.86 and 1.25%, respectively, indicating excellent precision and operational stability of the sensor.

**Figure 7:**
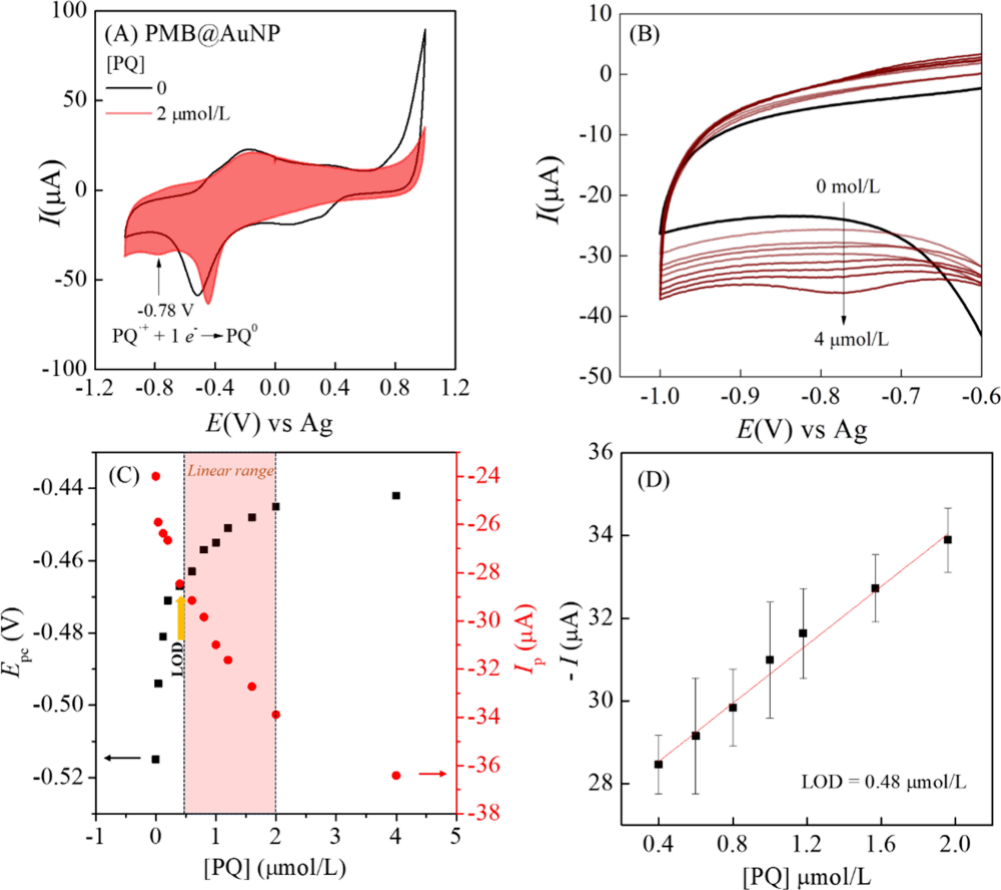
(A) Cyclic voltammograms of PMB@AuNP/SPCE recorded in the absence and presence of 2.0 × 10^–6^ mol/L paraquat. (B) Cyclic voltammograms with successive additions of paraquat in the range 4.0 × 10^–8^ to 4.0 × 10^–6^ mol/L. (C) Variation of peak potential and cathodic current as a function of paraquat concentration, data from CVs in Figure 7B. (D) Calibration plot of the cathodic peak current at −0.78 V within the linear concentration range of paraquat (6.0 × 10^–7^ to 4.0 × 10^–6^ mol/L).

Considering real sample analysis as a key factor to evaluate the practical applicability of the sensor, PQ detection was performed on river water samples using the standard addition method (Figure S7). The analysis exhibited a linear response over the investigated concentration range from 1.0 × 10^–6^ to 4.0 × 10^–6^ mol/L in the presence of 0.1 mol/L KCl, obtaining a recovery value between 88.6 and 97.6% (Table 2). These results fall within the acceptable RSD range, as international guidelines for pesticide residue analysis establish that mean recoveries at the relevant concentration levels should range from 70 to 120%, with precision not exceeding 20% RSD for environmental samples,^30^ underscoring the ongoing challenges associated with pesticide residue analysis. However, a lower recovery of 74.0% was observed at a concentration of 4.0 × 10^–6^ mol/L. This decrease may be attributed not only to matrix interference but also to the adsorption of PQ species onto the electrode surface, which can influence the effective electrochemical response at higher concentrations.

**Table 2:** Results of the PQ Recovery Tests Obtained by Electrochemical Sensing Using PMB@AuNP/SPCE

**spiked concentration (μmol/L)**	**recovery (%)**	**RSD (%)**
1.0	97.6	6.1
1.6	91.7	1.7
2.0	88.6	0.6
4.0	74.0	0.1

Indeed, an interesting finding is that the potential shift limit of the PQ^+^· species coincides with the onset of the linear response for PQ^0^. This intersection corresponds to the limit of detection (LOD) and marks the beginning of the linear detection range for PQ, as highlighted in Figure 7B. It suggests that the adsorption of PQ^+^· enhances the detection of PQ^0^. However, only the adsorbed species effectively contribute to the cathodic detection current. The adsorption of PQ-reduced species was also observed for other modified electrodes carrying out PQ detection by voltammetric techniques.^31^ Thus, coupling Raman analysis with voltammetric detection can help detect PQ in solution without the adsorption effect.

### 3.5 Exploratory Raman Spectroelectrochemical Analysis

The hybrid PMB@AuNP/SPCE electrode was evaluated as a potential spectroelectrochemical sensing platform, where the electrochemical properties can help enhance the sensitivity of the sensor and the Raman can help to enhance the selectivity. The Raman spectra collected at the PMB@AuNP/SPCE electrode surface without any solution and at OCP show the characteristic Raman spectra of MB molecules, as discussed in Figure 4. However, only the fluorescence background of the electrode was observed in 0.1 M KCl supporting electrolyte. Thus, the spectroelectrochemical properties of the PMB@AuNP film were evaluated in inert electrolyte, showing a fluorescence dependence with a shift to a lower wavenumber upon application of a negative potential (Figure S8) in potentiostatic mode.

PQ showed a peak at 1536 cm^–1^, ascribed to −CH_2_ bending and C–C stretching, when applying a potential of −0.73 V using an unmodified SPCE as reported by ref 32, but this also depends on the setup conditions. Thus, the negative potential can be used to enable selective detection of PQ in a mixture of similar compounds, such as diquat molecules.^32^ In our system, at OCP, no Raman signal from PQ was observed. However, upon applying a negative potential of −0.78 V, which is *E*_pc_ from cyclic voltammetry, a Raman band appeared at 1530 cm^–1^ (CH_2_ bending and C–C stretching) (Figure 8A). This electrochemical and spectroscopic response was observed for both unmodified SPCE and PMB@AuNP/SPCE and can be ascribed to the adsorption of the reduced radical species (PQ^+^·) on the electrode surface. Additionally, the MB fluorescence background decreases upon application of −0.78 V in the presence of PQ, which agrees with the results observed in inert electrolyte.

**Figure 8:**
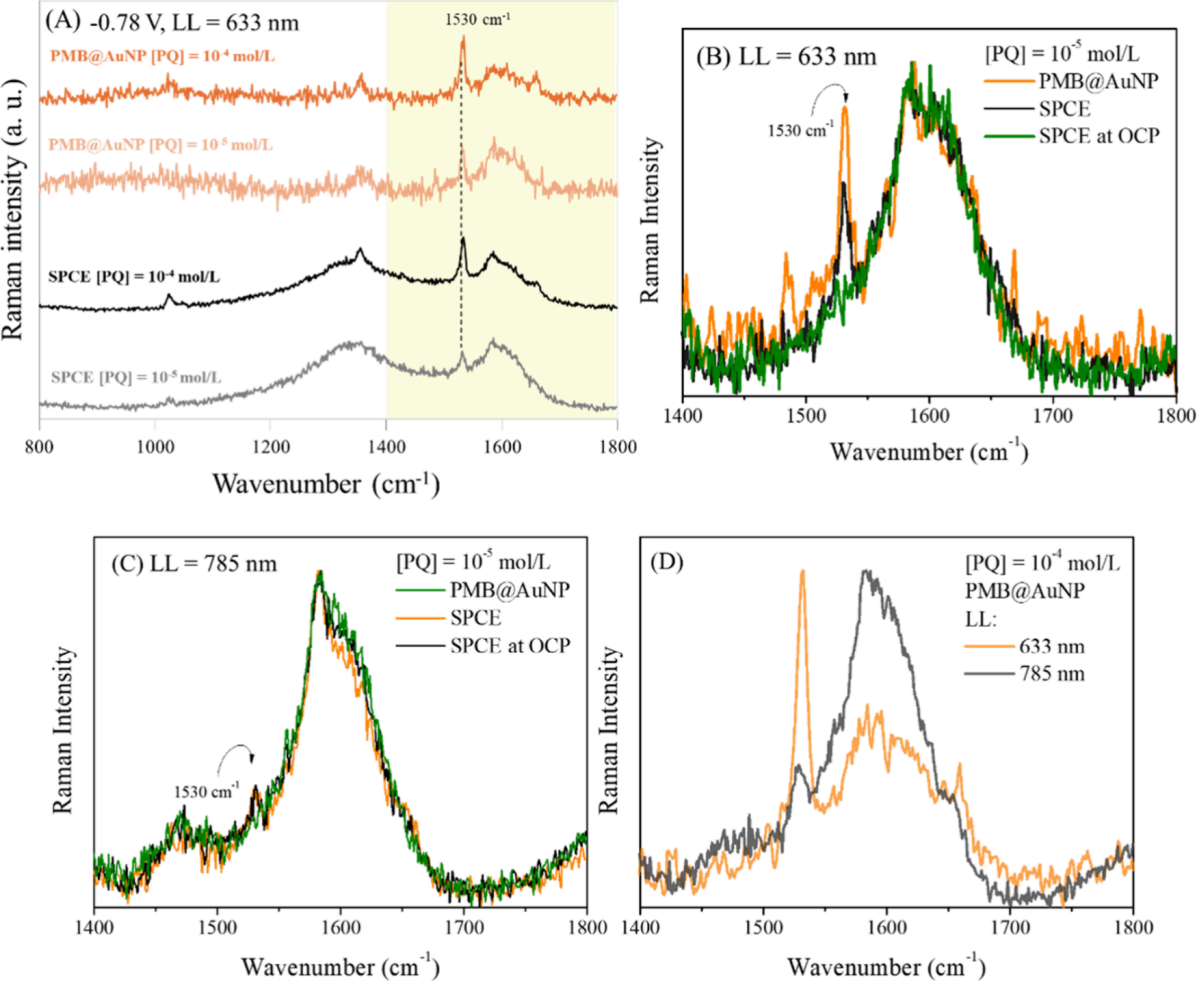
(A) Raman spectra of the unmodified SPCE and modified with PMB@AuNP film in the presence of 10^–4^ and 10^–5^ mol/L PQ applying −0.78 V, using the 633 nm laser line. (B, C) Comparison between the Raman spectra (1400–1800 cm^–1^) obtained at applied potential −0.78 V and at OCP at the unmodified SPCE and at the PMB@AuNP-modified SPCE, in the presence of 10^–5^ mol/L PQ, using the (B) 633 nm and (C) 785 nm laser lines. (D) Comparison between the Raman spectra (1400–1800 cm^–1^) obtained at PMB@AuNP/SPCE at applied potential −0.78 V, in the presence of 10^–4^ mol/L PQ, using the 633 and 785 nm laser lines. All Raman spectra were normalized to zero at 284 cm^–1^ with baseline correction.

A higher signal-to-noise (S/N, see the Supporting Information (SI)) ratio 54 vs 18 was observed for the PMB@AuNP film compared to the unmodified SPCE, indicating that the film improves the Raman signal under 633 nm laser excitation (Figure 8B). However, the enhanced factor (EF, see the SI) showed a value of only 2.9, even in the presence of gold nanoparticles, suggesting that only a modest Raman signal amplification is achieved under the investigated conditions.

The 785 nm laser measurements did not show significant changes at a PQ concentration of 10^–5^ M when the PMB@AuNP/SPCE was compared to the unmodified SPCE at −0.78 V applied potential (Figure 8C). Additionally, the PMB@AuNP/SPCE exhibited a signal three times lower than the response obtained using the 633 nm laser. This difference can be attributed to the resonance between the 633 nm laser and the absorption spectrum of the PMB@AuNP film, which is associated with the electronic transitions of the methylene blue (MB) species. The visible absorption spectra of these films deposited on an ITO electrode are shown in Figure S9, displaying absorption bands corresponding to the MB monomer and dimer at 630 and 680 nm, respectively. Therefore, the spectroelectrochemical studies, which vary the laser wavelength, suggest that better responses are achieved when resonance conditions are met.

Spectroscopic measurements performed at PQ concentrations below 10^–5^ mol/L did not exhibit a signal-to-noise ratio sufficient for reliable spectral interpretation and determination of the LOD. This observation reinforces that, under the experimental conditions employed, no measurable SERS effect is achieved, the enhancement factor being only 2.9, even in the presence of gold nanoparticles. This suggests that the observed response is due to modulation of the Raman signal induced by the applied potential of −0.78 V. The absence of a significant SERS response suggests that plasmonic enhancement was either negligible or masked by the fluorescence background and interfacial electronic effects, thereby limiting the quantitative applicability of the spectroscopic technique at low analyte concentrations. In contrast, electrochemical measurements provided superior analytical reliability, enabling sensitive and reproducible detection of PQ, using the spectroscopic signal to improve the selectivity. Thus, the coupling of electrochemical and spectroscopic techniques remains advantageous, as it offers an additional level of selectivity and analytical confidence. In particular, the spectroscopic signal associated with PQ was observed exclusively when the characteristic reduction potential was applied, reinforcing the assignment of the detected species and supporting the electrochemical identification of the analyte at the electrode interface.

## 4 Conclusions

The hybrid PMB@AuNP films demonstrated a strong dependence of their effective surface area on the molar ratio used during their formation by electrodeposition. As expected, the incorporation of gold nanoparticles contributed to an increase in surface roughness and contact area, enhancing electrochemical activity without interfering with the electrodeposition mechanism of PMB. This structural modification directly influenced the charge transfer process, following the trend observed for the effective surface area. The PMB@AuNP (2:1) film-modified SPCE exhibited the most balanced charge transfer behavior, combining increased surface area and favorable electrostatic interactions. These findings provide valuable insights into the potential of PMB@AuNP film-modified electrodes for real-time spectroelectrochemical monitoring and sensing applications. The combined electrochemical and optical responses expand the possibilities for developing advanced detection strategies in environmental sensing, being dependent on the resonance conditions of the film with a laser line (in this case, a laser line at 633 nm). Additionally, cyclic voltammetry measurements revealed a detection limit of 0.48 μmol/L for paraquat in water and a recovery of 88.6–97.6% for water river samples, demonstrating the practical applicability of these hybrid films for detecting low levels of paraquat. These features highlight the potential of PMB@AuNP-modified SPCEs as versatile platforms for integrated electrochemical and spectroelectrochemical sensing.

## Supplementary Material



## Notes

Additional electrochemical, spectroscopic, and morphological data are provided, including: The Supporting Information is available free of charge at https://pubs.acs.org/doi/10.1021/acsomega.6c00888.

Cyclic voltammetry for PMB electrodeposition (5, 10, and 20 cycles) and scan rate studies (5–300 mV s^–1^) in [Fe­(CN)_6_]^3–^/[Fe­(CN)_6_]^4–^, including peak current vs square root of scan rate relationships; cyclic voltammograms of the first potential cycle for PMB and PMB@AuNP (2:1) on SPCE are included; EIS Nyquist plots for PMB@AuNP-modified SPCE (10:1, 4:1, and 2:1) were recorded in [Fe­(CN)_6_]^3–^/[Fe­(CN)_6_]^4–^ containing 0.1 mol L^–1^ KCl; cyclic voltammetry in river water samples spiked with paraquat (1.0–4.0 μmol L^–1^); Raman spectra (λ = 633 nm) at different applied potentials and UV–vis spectra of films on ITO electrodes; and signal-to-noise ratio (S/N) and enhancement factor (EF) calculations (PDF)
