# Supplementary material for: Electrochemically Controlled Raman Response in Electropolymerized Polymethylene Blue–Gold Nanoparticle Films on Screen-Printed Carbon Electrodes
Source: ACS Omega. 2026 Mar 30;11(13):21357–68. doi: 10.1021/acsomega.6c00888 (PMC13063051; doi:10.1021/acsomega.6c00888)
Supplement: Supplementary file 1 [file ao6c00888_si_001.pdf]

**Supporting Information**

# Electrochemically Controlled Raman Response in Electropolymerized Polymethylene Blue–Gold Nanoparticle Films on Screen-Printed Carbon Electrodes


Azeneth Borja[1], Fernando A. Basso[1], Henry S. Kavazoi[2], Christopher M. A. Brett[3], Priscila Aléssio[2], Cibely S. Martin[1*]

[1] São Paulo State University (UNESP), School of Engineering, Ilha Solteira-SP, 15385-007, Brazil.
[2]São Paulo State University (UNESP), School of Sciences and Technology, Presidente Prudente-SP, 19060-080, Brazil.
[3]Department of Chemistry, CEMMPRE, ARISE, Faculty of Sciences and Technology, University of Coimbra, 3004-535 Coimbra, Portugal
*Corresponding author: cibely.martin@unesp.br


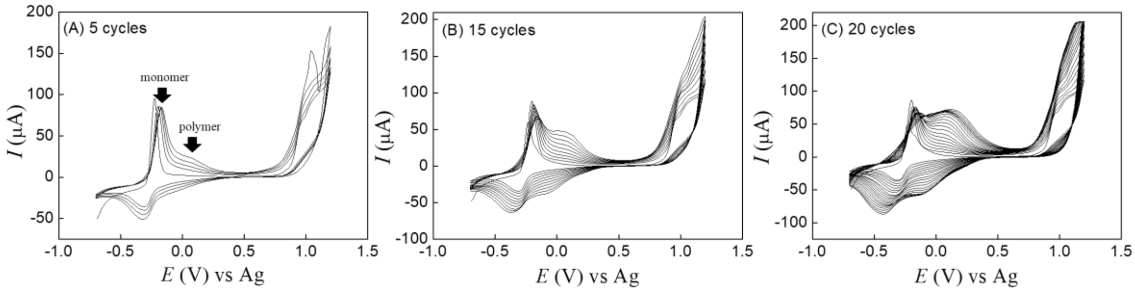


**Figure S1**. Cyclic voltammetry for electrodeposition of PMB during 5, 10, and 20 potential cycles.

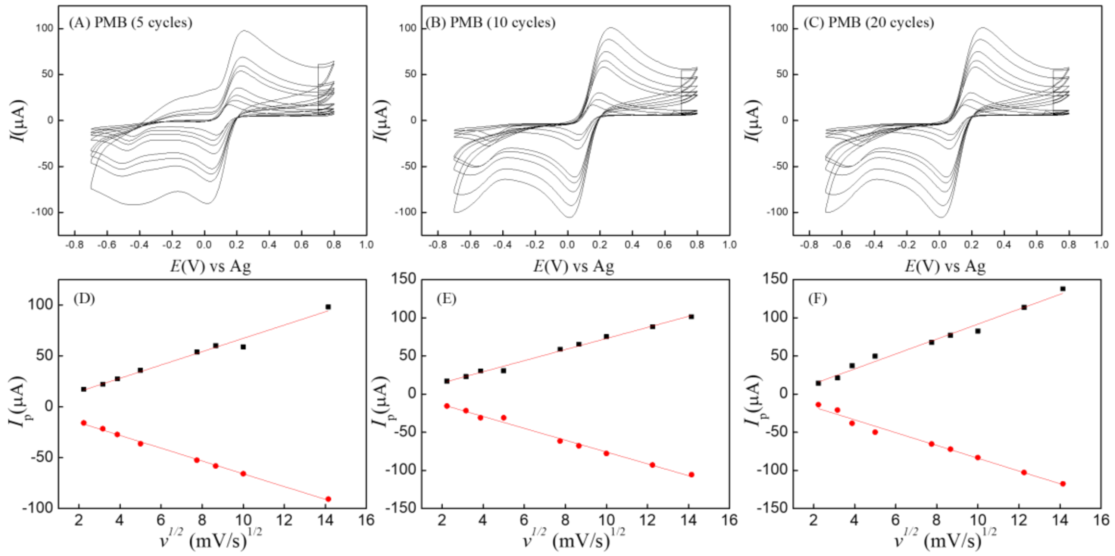


**Figure S2.** Cyclic voltammograms obtained from 5 to 300 mV/s in 2 mmol/L $[Fe(CN)_6]^{3-}$ /$[Fe(CN)_6]^{4-}$ for PMB electrodeposited with (A) 5, (B) 10, and (C) 20 potential cycles. Relation of peak current and the square root of scan rate for PMB electrodeposited with (D) 5, (E) 10, and (F) 20 potential cycles.

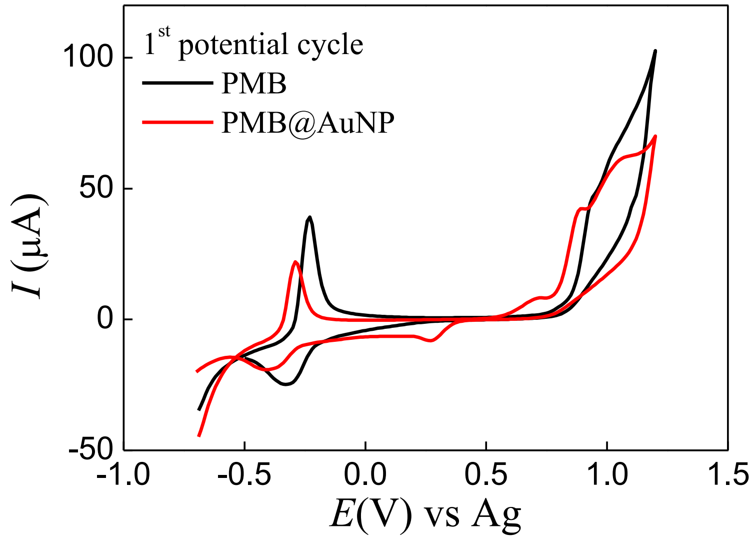


**Figure S3.** Cyclic voltammograms of the first potential cycle during the electropolymerization of PMB and PMB@AuNP (2:1) on SPCE.

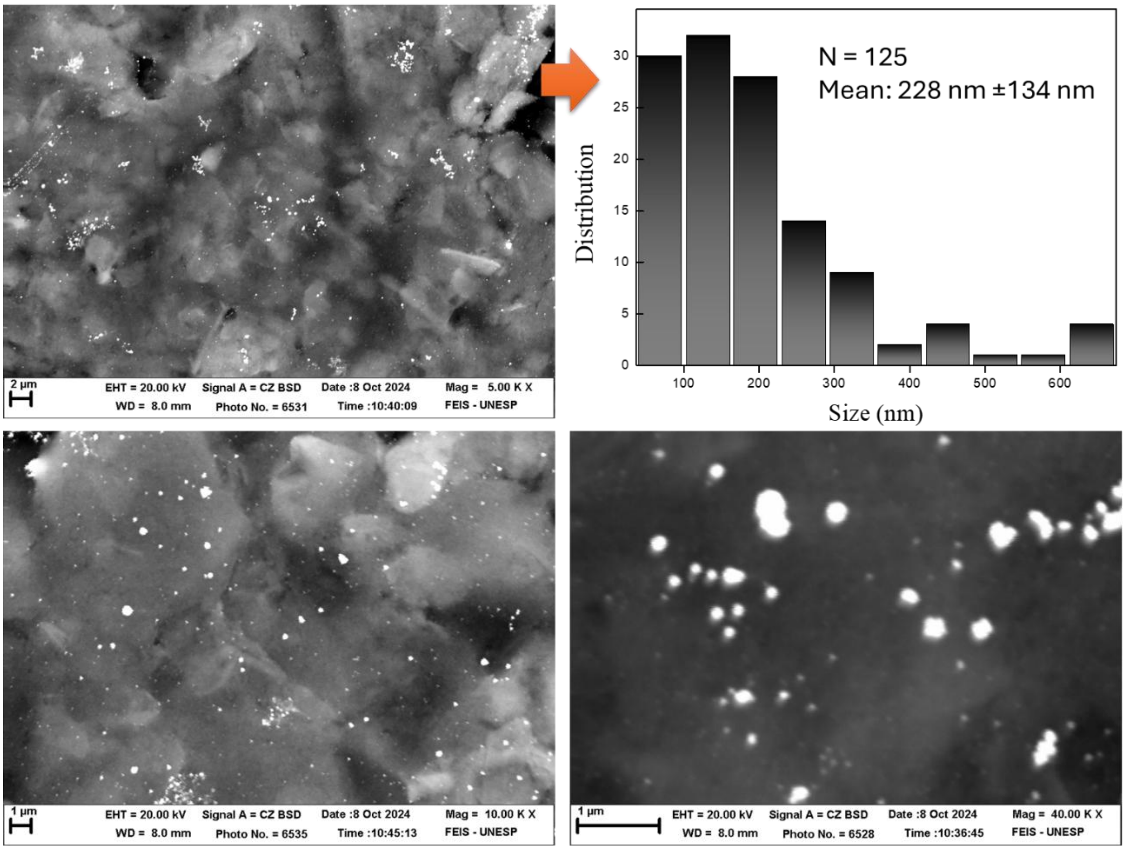


**Figure S4.** SEM images for PMB@AuNP film and size distribution obtained by using ImageJ free software.

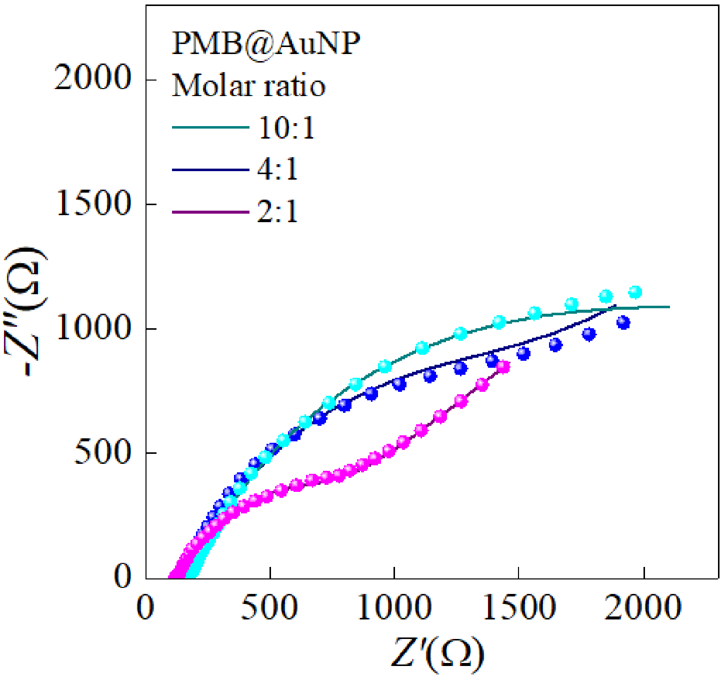


**Figure S5.** Complex plane impedance spectra (Nyquist plots) for SPCE modified with PMB@AuNP electrodeposited with different molar ratios (10:1; 4:1. 2:1). EIS measurements carried out at OCP in $[Fe(CN)_6]^{3-}/[Fe(CN)_6]^{4-}$ redox probe solution containing 0.1 mol/L KCl.

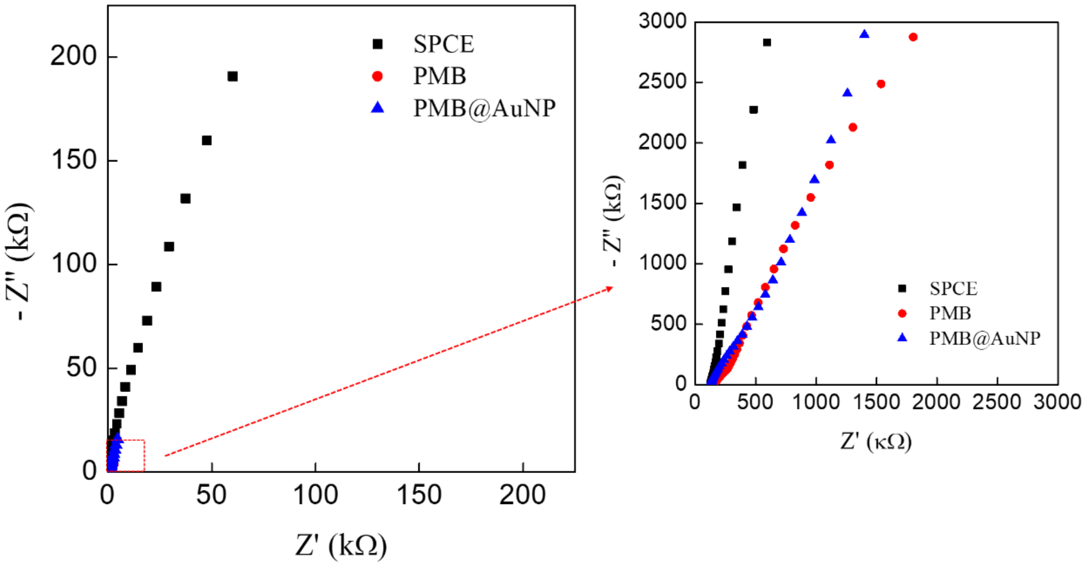


**Figure S6.** Complex plane impedance spectra (Nyquist plots) obtained for SPCE unmodified and modified with PMB (2:1). EIS measurements carried out at OCP in $[Ru(NH_3)_6]^{2+}$ redox probe solution containing 0.1 mol/L KCl.

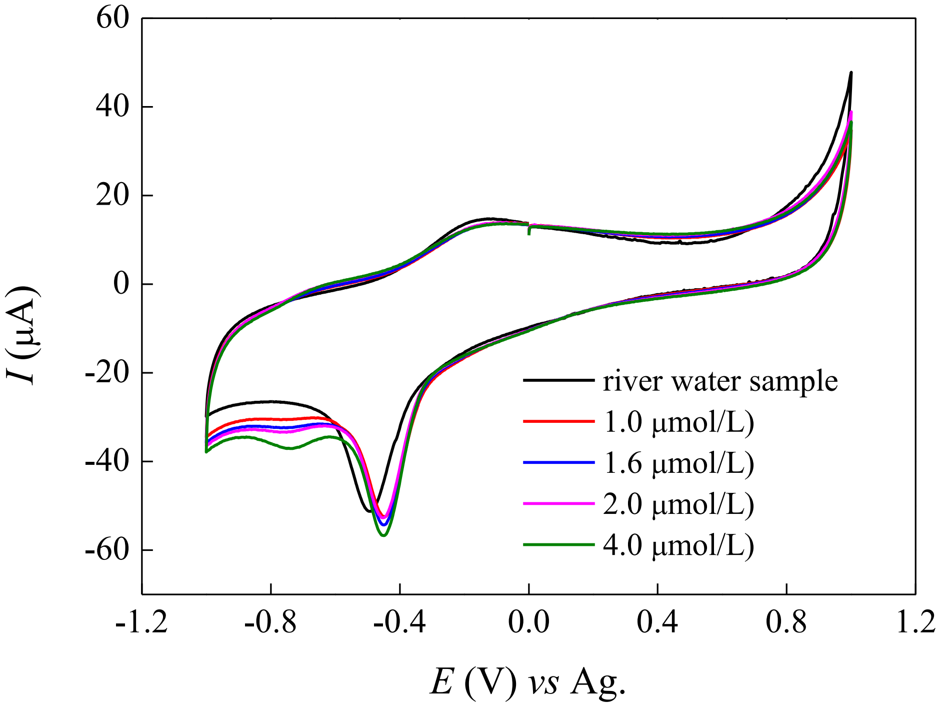


**Figure S7.** Cyclic voltammogram recorded with river water samples with addition of 1.0 to 4.0 µmol/L of PQ standard solution in 0.1 mol/L KCl. $v$ = 50 mV/s.

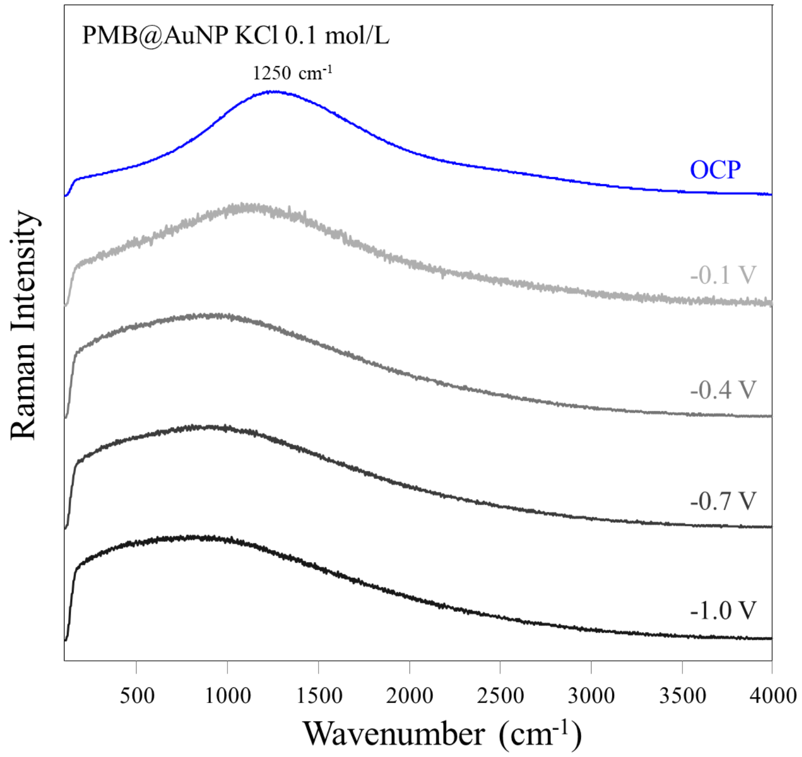


**Figure S8**. Raman spectra collected at PMB@AuNP/SPCE surface applying the indicated negative potentials. Laser line: 633 nm.

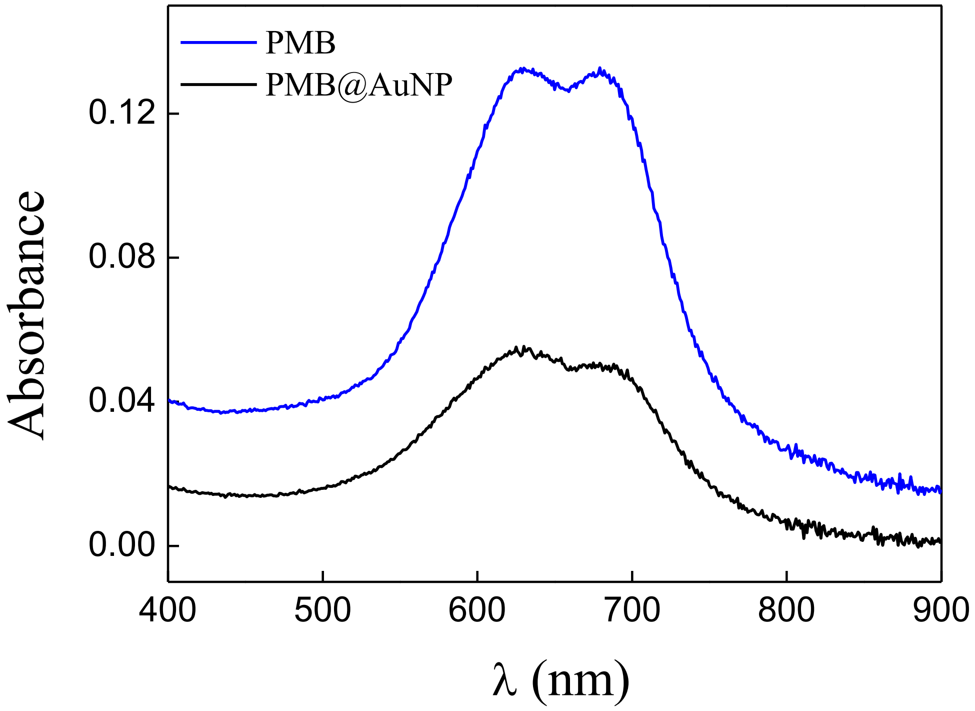


**Figure S9**. Visible spectra of PMB and PMB@AuNP films deposited onto ITO electrodes. The films were deposited using the same conditions as for SPCE electrode substrates.

**Calculation of signal-to-noise (S/N) and enhancement factor (EF) from Raman measurements**

*Signal-to-noise (S/N)*

$$S/N = \frac{I_{sample} - I_{blank}}{\sigma} \qquad (S1)$$

**Measurements carried out at OCP**

**Blank signal** (**$I_{blank}$**) = 44 counts

**noise (σ)** = 9.2 (standard deviation from blank signal)

**Measurements carried out at -0.78V**

**SPCE signal (**$I_{sample}$**)** = 214 counts

**PMB@AuNP/SPCE signal (**$I_{sample}$**)** = 540 counts

*Enhancement factor (EF) considering the same paraquat concentrations ($10^{-4}$ mol/L)*

$$EF = \frac{I_{modified} - I_{blank}}{I_{unmodified} - I_{blank}} \qquad (S2)$$

**Measurements carried out at OCP**

**Blank signal** (**$I_{blank}$**) = 44 counts

**SPCE signal (**$I_{unmodied}$**)** = 214 counts

**PMB@AuNP/SPCE (**$I_{modified}$**)** = 540 counts
